# Statistical assessment of the global regulatory role of histone acetylation in *Saccharomyces cerevisiae*

**DOI:** 10.1186/gb-2006-7-8-r70

**Published:** 2006-08-02

**Authors:** Guo-Cheng Yuan, Ping Ma, Wenxuan Zhong, Jun S Liu

**Affiliations:** 1 Department of Statistics, Harvard University, Cambridge, MA 02138, USA; 2 Bauer Center for Genomics Research, Harvard University, Cambridge, MA 02138, USA; 3 Department of Statistics, University of Illinois, Champaign, IL 61820, USA; 4 Institute for Genomic Biology, University of Illinois, Champaign, IL 61820, USA

## Abstract

**Background:**

Histone acetylation plays important but incompletely understood roles in gene regulation. A comprehensive understanding of the regulatory role of histone acetylation is difficult because many different histone acetylation patterns exist and their effects are confounded by other factors, such as the transcription factor binding sequence motif information and nucleosome occupancy.

**Results:**

We analyzed recent genomewide histone acetylation data using a few complementary statistical models and tested the validity of a cumulative model in approximating the global regulatory effect of histone acetylation. Confounding effects due to transcription factor binding sequence information were estimated by using two independent motif-based algorithms followed by a variable selection method. We found that the sequence information has a significant role in regulating transcription, and we also found a clear additional histone acetylation effect. Our model fits well with observed genome-wide data. Strikingly, including more complicated combinatorial effects does not improve the model's performance. Through a statistical analysis of conditional independence, we found that H4 acetylation may not have significant direct impact on global gene expression.

**Conclusion:**

Decoding the combinatorial complexity of histone modification requires not only new data but also new methods to analyze the data. Our statistical analysis confirms that histone acetylation has a significant effect on gene transcription rates in addition to that attributable to upstream sequence motifs. Our analysis also suggests that a cumulative effect model for global histone acetylation is justified, although a more complex histone code may be important at specific gene loci. We also found that the regulatory roles among different histone acetylation sites have important differences.

## Abstract 2

An analysis of genome-wide histone acetylation data using a few complementary statistical models gives support to a cumulative effect model for global histone acetylation.

## Background

Gene activities in eukaryotic cells are concertedly regulated by transcription factors and chromatin structure. The basic repeating unit of chromatin is the nucleosome, an octamer containing two copies each of four core histone proteins. Recent microarray based studies [1-8] have begun to uncover the global regulatory role of nucleosome positioning and modifications. While nucleosome occupancy in promoter regions typically occludes transcription factor binding, thereby repressing global gene expression [1-8], the role of histone modification is more complex [9-11]. Histone tails can be modified in various ways, including acetylation, methylation, phosphorylation, and ubiquitination. Even the regulatory role of histone acetylation, the best characterized modification to date, is still not fully understood [12,13].

Each of the four core histones contains several acetylable sites at their amino terminus tails. Genome-wide histone acetylation data from *Saccharomyces cerevisiae *[2,8] have offered new opportunities for us to evaluate the regulatory effects of histone acetylation at these lysine sites. In particular, both H3 and H4 acetylation levels were found to be positively correlated with gene transcription rates. However, a subtle but important issue in analyzing such data is that effects of other potentially important factors not included in the analysis, generally termed as confounding factors, cannot be revealed by simple correlation plots. It is unclear, for example, how much regulatory information associated with histone acetylation is redundant with the genomic sequence information. To gain insights into this, we conducted a statistical analysis by combining acetylation [2,4,8], nucleosome occupancy [1,3,8], gene upstream sequence information [14], and gene expression data [1,15,16] to investigate the effect of histone acetylation in the context of other regulatory factors in *S. cerevisiae*.

A related question is whether different histone acetylation sites play similar roles in gene regulation. It is commonly postulated that globally H3 and H4 acetylation are both associated with global gene activation. Indeed, the acetylation levels of H3 and H4 across gene promoters have been shown to be highly correlated [2,7,8]. However, other experimental studies have also suggested that H3 and H4 acetylations have different regulatory roles [17-20]. We investigated the validity of a cumulative model for the regulatory effect of histone acetylation and also compare the regulatory effects of H3 and H4 acetylation in a coherent statistical framework.

Another interesting question is whether combinatorial patterns of histone acetylation code for distinct regulatory information at a global level [9,11], with each pattern being recognized by a specific regulatory protein. If such codes exist, a large number of codes may result from combinations of different histone acetylation sites. On the other hand, if the effect is cumulative, multiple histone acetylation sites may be used to gradually control the interaction between nucleosomes or the stability of the regulatory proteins. Recent mutagenesis studies [5] have suggested that multiple H4 acetylation sites have a cumulative effect. Here we revisit this question using a statistical approach to combine available genome-wide data. Our analysis suggests that the simple additive-effect model is sufficient for fitting the available data.

## Results

### Effect of histone acetylation on gene transcription rate

#### Standard analysis

We analyzed two recent genome-wide histone acetylation datasets [2,8] (see Materials and methods for details about the data sources). Due to space limits, here we only present the results for Pokholok *et al*.'s data [8], with the discussion of Kurdistani *et al*.'s data [2] in Additional data file 1. Pokholok *et al*. measured acetylation levels at three different sites, H3K9, H3K14, and H4, with the last referring to non-specific acetylation on any of the four acetylable lysines on H4 tails.

A typical analysis, when both histone acetylation data on a single site (for example, H3K9) and transcription rate data are available, is to simply correlate the two sets of measurements and to report the apparent significant statistical correlation between the two. When data on multiple acetylation sites are available, a slightly more formal analysis is to fit a linear regression model of the form:

yi=α+∑jβjxij+εi,     (equation 1)
 MathType@MTEF@5@5@+=feaafiart1ev1aaatCvAUfeBSjuyZL2yd9gzLbvyNv2Caerbhv2BYDwAHbqedmvETj2BSbqee0evGueE0jxyaibaiKI8=vI8tuQ8FMI8Gi=hEeeu0xXdbba9frFj0=OqFfea0dXdd9vqai=hGuQ8kuc9pgc9s8qqaq=dirpe0xb9q8qiLsFr0=vr0=vr0dc8meaabaqaciGacaGaaeqabaqadeqadaaakeaacaWG5bWaaSbaaSqaaiaadMgaaeqaaOGaeyypa0JaeqySdeMaey4kaSYaaabuaeaacqaHYoGydaWgaaWcbaGaamOAaaqabaaabaGaamOAaaqab0GaeyyeIuoakiaadIhadaWgaaWcbaGaamyAaiaadQgaaeqaaOGaey4kaSIaeqyTdu2aaSbaaSqaaiaadMgaaeqaaOGaaiilaiaaxMaacaWLjaWaaeWaceaacaqGLbGaaeyCaiaabwhacaqGHbGaaeiDaiaabMgacaqGVbGaaeOBaiaabccacaaIXaaacaGLOaGaayzkaaaaaa@51BB@

where *y*_*i *_is the transcription rate of gene *i*, and *x*_*ij*_, for *j *= 1,2,3, is the histone acetylation level of H3K9, H3K14, and H4, respectively. All data were log-transformed before analysis. This model is highly statistically significant for both intergenic (*p *value < 2.0 × 10^-16^) and coding regions (*p *value < 2.0 × 10^-16^). The association between gene expression and intergenic histone acetylation is commonly interpreted as regulatory effects, whereas correlation between gene expression and coding histone acetylation is believed to be a result of passing of transcriptional machineries through active genes.

#### Significant confounding factors

Gene regulation is a complex process involving many contributing factors. Probably the best characterized factor for controlling gene transcription is the upstream sequence information. Although histone acetyltransferases (HATs) and histone deacetylases (HDACs) do not have obvious sequence specificity themselves, they may be recruited by transcription factors that recognize specific sequences. Thus, sequence information is an important confounding factor. Our main interest here is to delineate the roles of these factors and investigate whether histone acetylation provides any additional information on gene transcription. In the past decade, numerous computational methods have been developed to identify target sequences of transcription factors and to use such information to predict gene expression [21-26].

Another well-characterized property of the chromatin structure, the nucleosome occupancy, also plays an important role in gene regulation. Histone acetylation and nucleosome positioning are closely related events. Genome-scale, high-resolution nucleosome positioning data have led to the observation that transcription factor binding sites tend to be nucleosome-depleted [6]. Although genome-wide, high-resolution nucleosome positioning data are still unavailable, lower resolution data have already shown that gene expression levels are reciprocally correlated with nucleosome occupancy [1,3,8]. Therefore, nucleosome occupancy may also be an important confounding factor in explaining gene regulation.

#### Refined analysis

We tested using two different sequence motif based-methods to account for the *cis *regulatory information (see Materials and methods for details). As shown in Additional data file 1, the two methods gave remarkably consistent results. Here we present results from using MDscan [24], which infers sequence motif information *de novo*. The combined transcriptional control by transcription factor binding motifs (TFBMs), nucleosome occupancy, and histone acetylation is modeled as:

yi=α+∑jβjxij+∑jηjzij+δwi+εi,     (equation 2)
 MathType@MTEF@5@5@+=feaafiart1ev1aaatCvAUfeBSjuyZL2yd9gzLbvyNv2Caerbhv2BYDwAHbqedmvETj2BSbqee0evGueE0jxyaibaiKI8=vI8tuQ8FMI8Gi=hEeeu0xXdbba9frFj0=OqFfea0dXdd9vqai=hGuQ8kuc9pgc9s8qqaq=dirpe0xb9q8qiLsFr0=vr0=vr0dc8meaabaqaciGacaGaaeqabaqadeqadaaakeaacaWG5bWaaSbaaSqaaiaadMgaaeqaaOGaeyypa0JaeqySdeMaey4kaSYaaabuaeaacqaHYoGydaWgaaWcbaGaamOAaaqabaaabaGaamOAaaqab0GaeyyeIuoakiaadIhadaWgaaWcbaGaamyAaiaadQgaaeqaaOGaey4kaSYaaabuaeaacqaH3oaAdaWgaaWcbaGaamOAaaqabaaabaGaamOAaaqab0GaeyyeIuoakiaadQhadaWgaaWcbaGaamyAaiaadQgaaeqaaOGaey4kaSIaeqiTdqMaam4DamaaBaaaleaacaWGPbaabeaakiabgUcaRiabew7aLnaaBaaaleaacaWGPbaabeaakiaacYcacaWLjaGaaCzcamaabmGabaGaaeyzaiaabghacaqG1bGaaeyyaiaabshacaqGPbGaae4Baiaab6gacaqGGaGaaGOmaaGaayjkaiaawMcaaaaa@602F@

where the *x*_*ij *_values are the three histone acetylation levels (corresponding to H3K9, H3K14, and H4, respectively), the *z*_*ij *_values are the corresponding scores to the 33 selected motifs, and *w*_*i *_is the nucleosome occupancy level. Table 1 shows the R^2 ^(referring to the adjusted R-square statistic, which measures the fraction of explained variance after an adjustment of the number of parameters fitted; see page 231 of [27]) of the various linear models. One can see that a simple regression of transcription rates against histone acetylation without considering any other factors gave an R^2 ^of 0.1841 (Table 1), implying that about 18% of the variation of the transcription rates is attributable to histone acetylation. In contrast, the regression of transcription rates against motif scores and nucleosome density levels (no histone acetylation) gave an R^2 ^of 0.1997. The comprehensive model with all the variables we considered (equation 2) bumped up the R^2 ^to 0.3262, indicating that the histone acetylation does have a significant effect on the transcription rate, although not as high as that in the naïve model.

**Figure 1:**
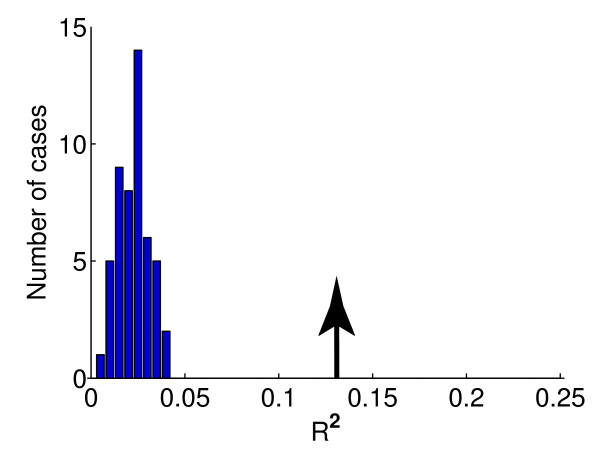
Model validation by comparing the R^2 ^for the real versus randomly permutated datasets. The R^2 ^obtained by applying the motif selection and fitting equation 2 (with sequence motif information only) procedures to randomly permutated and real data. The histogram is obtained based on 50 randomly permutated samples. The arrow on the right marks the R^2 ^for the real data. Results for the coding regions are represented here. See the main text for details.

To confirm that the above results indicate intrinsic statistical associations rather than artifacts of the statistical procedure, we validated our model using two independent methods. First, we tested whether applying the above procedure to the random inputs would yield substantially worse performance than applying it to the real data. We generated 50 independent samples by random permutation (see Materials and methods). The R^2 ^for these randomized data are much smaller than for the real data. For example, considering equation 2 fitted with sequence motif information only, the largest R^2 ^for coding regions for the 50 randomly permutated samples is 0.0378 (Figure 1), compared to R^2 ^of 0.1315 for the real data. The differences between the R^2 ^values are even larger if we also include histone acetylation and nucleosome occupancy in the model. Therefore, our model is able to extract real statistical association. Secondly, we tested whether the model might overfit by a five-fold cross validation procedure (see Materials and methods). The root mean square (rms) errors for the training data are 1.500 (for intergenic regions) and 1.483 (for coding regions), whereas the rms errors for the testing data were 1.519 (for intergenic regions) and 1.498 (for coding regions). In both cases, the difference between the in-sample and out-of-sample errors is less than 2%, suggesting overfitting is not an issue here.

### Multiple histone acetylation sites have cumulative regulatory effects

With the foregoing regression framework, we further investigated whether the combined effect of various histone acetylation sites could be approximated by a simple cumulative model, or a more complex 'combinatorial histone code' is needed. To gain a qualitative overview, we grouped genes according to their upstream histone acetylation patterns, each corresponding to a combination of high (greater than 60th percentile) or low (less than 40th percentile) histone acetylation levels at three acetylation sites. To avoid ambiguity due to measurement noise, the middle 20% of genes was not included in any groups. This coarse-grained partition method results in eight groups of genes with distinct upstream acetylation patterns. For example, one of these eight groups contains genes with high H3K9, high H3K14, and low H4 acetylation levels in their upstream intergenic regions. Increasing H3K9 acetylation level enhances gene transcription (Table 2), regardless of the acetylation level at other sites. A similar but weaker pattern can be seen for H3K14 acetylation. In contrast, the increase of H4 acetylation level is associated with both elevated and reduced transcription rates. A possible explanation is that the regulatory effect of H4 acetylation is dependent on acetylation level at other sites, while another explanation is that the H4 acetylation effect is weak overall. These relationships are not sensitive to the cutoff threshold for removing ambiguous genes.

As a quantitative validation of the above observation, we re-examined the validity of equation 2 in modeling the regulatory role of histone acetylation. We observed that the inclusion of all quadratic interaction terms among the three histone acetylation covariates in the regression model does not improve the model fitting (that is, R^2 ^= 0.3262 and 0.3278, respectively, for intergenic regions, and R^2 ^= 0.3131 and 0.3132, respectively, for coding regions). The same conclusion also holds when we do not include the sequence motif information and nucleosome occupancy data as covariates (R2 = 0.1841 and 0.1925, respectively, for intergenic regions). These observations suggest that the combinatorial effect is, at best, undetectable from the current data and the simple cumulative model (equation 2) is sufficient. Similar results were obtained using the acetylation data in Kurdistani *et al*. [2] (Additional data file 1).

### H3 and H4 acetylation play different roles in gene regulation

**Figure 2:**
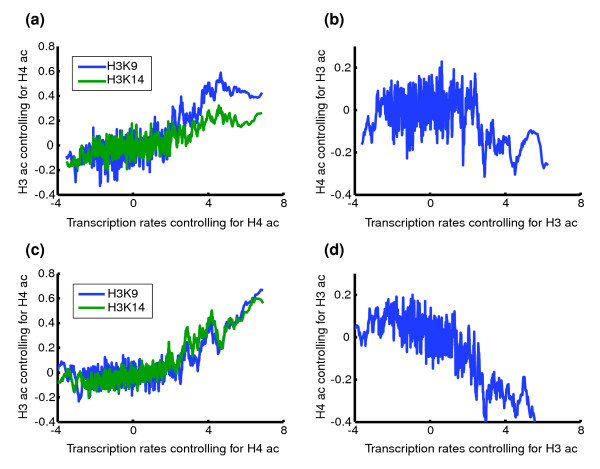
Dependency of transcription rates on histone acetylation levels (ac) after controlling for confounding effects. **(a) **Transcription rates versus intergenic H3K9 and K14 acetylation levels controlling for H4 acetylation levels. **(b) **Transcription rates versus intergenic H4 acetylation levels controlling for H3K9 and K14 acetylation levels. **(c) **Same as (a) except that coding region histone acetylation data are used. **(d) **Same as (b) except that coding region histone acetylation data are used. All data are log-transformed. Genes are sorted by transcription levels. A sliding smoothing window of 20 genes is applied to the transcription rates and histone acetylation data.

A statistical measure of the effect of an individual factor/covariate on the response variable is the partial correlation (Materials and methods), which roughly reflects the 'pure' relationship between two variables while controlling other factors. As shown in Table 3, partial correlations between transcription rates and intergenic H3K9 and H3K14 acetylation levels, while controlling the sequence information and H4 acetylation levels, are 0.25 and 0.21, respectively; whereas that between transcription rates and H4 acetylation effect is insignificant (-0.03). In addition, the difference between the effects of H3 and H4 acetylations is visually evident (Figure 2). The same phenomenon can be observed by comparing different regression models. As shown by Table 1, the R^2 ^(adjusted R-square) for the model without using the H4 acetylation information is comparable to the full model, whereas the performance of the model without H3 acetylation is significantly poorer. Interestingly, the transcription rate is negatively correlated with coding region H4 acetylation. These observations suggest that while H3 acetylation plays an important role in global gene activation, H4 acetylation in the intergenic region has little global effect. Similar results were also obtained for the acetylation data in Kurdistani *et al*. (Additional data file 1).

Gcn5 is the catalytic component of the SAGA complex and preferentially acetylates H3 lysines, including K9, K14, and K18. Esa1 is the catalytic component of the NuA4 complex and preferentially acetylates H4 lysines. Based on the above analysis, we predicted that the global gene expression was significantly affected by the abundance of Gcn5 but not of Esa1. Genome-wide occupancy of Gcn5 and Esa1 has been measured previously [4]. Both enzymes were found to be globally associated with active genes, and the Pearson correlation between the occupancy levels of the two is as high as 0.7890, probably because they share a common component, Tra1, for recognizing targets [28]. We modified equation 2 to estimate the association between Pol II occupancy [4] and these two HATs. In this case, the response variable *y*_*i *_is the log-ratio of Pol II binding, whereas the *x*_*ij *_(*j *= 1,2) are the log-transformed binding ratios of Gcn5 and Esa1, respectively. Fitting the model using both Gcn5 and Esa1 data yields an R^2 ^of 0.2365. If we remove Gcn5 from the model, the R^2 ^is reduced to 0.1808. However, removing Esa1 causes little change in model performance (R^2 ^= 0.2366). In addition, the partial correlation between the occupancy levels of Pol II and Gcn5 while controlling Esa1 occupancy is 0.2690, whereas the number is reduced to only 0.0154 if the order of Gcn5 and Esa1 is reversed. Taken together, these results show that, indeed, Esa1 only marginally affects the global association with Pol II binding.

## Discussion

The global regulatory role of histone acetylation is still not fully understood and contradictory results have been reported in the literature [2,5,8]. Part of this inconsistency has been attributed to data analysis procedures [29]. In this paper, we analyzed two recent CHIP-chip datasets [2,8] in a statistically coherent framework. Our model isolates the regulatory role of individual acetylation sites and systematically controls for the effects of important confounding factors, thus resulting in a more detailed evaluation of the regulatory role of histone acetylation than previous studies [2,8]. Interestingly, our analyses of the two aforementioned datasets yielded similar results, even though the biological interpretations in the original papers were drastically different.

We found that the regulatory effect of histone acetylation can be well approximated by a simple regression model. In contrast to Kurdistani *et al*.'s claims [2], our results suggest that the currently available data supports a simple cumulative effect model instead of a combinatorial code model of histone modifications as originally proposed in [9], consistent with a recent mutagenesis study [5] showing that three of the four acetylable sites on H4 tails are functionally redundant. It is worth noting that these results do not exclude the possibility that combinatorial control is critical at specific gene loci, but it is unlikely that a fully combinatorial code regulates global gene expression.

We also quantitatively analyzed the regulatory effects due to individual acetylation sites. To our surprise, we found that the overall effects of H3 and H4 acetylation were quite different, at least statistically. In particular, while elevated H3 acetylation in promoter regions appears to be responsible for activating global gene expression, H4 acetylation seems to play a less important role. Levels of H3 and H4 acetylation in intergenic regions are closely coordinated by the binding of Gcn5 and Esa1, both of which have been found to bind to actively transcribed genes [4]. However, our analysis suggests that Esa1 may not be important for global regulation, consistent with previous experimental studies by Kevin Struhl's group [17,30,31]. In these studies, the authors show that depletion of Esa1 causes a global decrease of H4 acetylation, but only a small subset of the genes responds with significant transcription change [30]. They also found that the effect of H4 acetylation may be highly transcription factor specific [17]. It will be interesting to further investigate whether there is any biological benefit for the co-recruitment of Esa1 and Gcn5 to activate genes.

Histone modification in coding regions is often viewed as demarcating recent transcriptional events rather than playing a regulatory role. In this view, our analysis suggests that, along with methylation, acetylation also serves as a potent marker for transcription activities. On the other hand, H4 acetylation in coding regions may also have important regulatory roles. For example, the binding of the HDAC protein Hos2 to coding regions is important for active transcription [18,20]. The negative partial correlation between transcriptional activities and H4 acetylation levels is consistent with the aforementioned experimental results.

## Materials and methods

### Data sources

Datasets analyzed in this study include those for histone acetylation, nucleosome occupancy, gene expression, and genome sequence. In two recently published papers, genome-wide histone acetylation levels at eleven [2] and three sites [8] in yeast were measured using CHIP-chip. A major difference in experimental procedure between these two studies is that the acetylated DNA was hybridized against nucleosomal DNA on microarrays in Pokholok *et al*.'s study [8], but was hybridized against the genomic DNA in Kurdistani *et al*.'s study [2]. Since in the latter dataset histone acetylation was confounded with nucleosome occupancy, our discussion in the main text is focused on analyzing Pokholok *et al*.'s data. To compare the results from the two experiments, we repeated our analysis procedure on a normalized version of Kurdistani *et al*.'s data after removing its dependency on nucleosome occupancy. We found that the main conclusions remain the same. Detailed analysis of Kurdistani *et al*.'s data is presented in Additional data file 1.

In addition, several groups have measured genome-wide nucleosome occupancy in yeast [1,3,8]. We chose to utilize Pokholok *et al*.'s nucleosome occupancy data in our analysis as well, since nucleosome occupancy has a clear effect on gene regulation [6]. We used Bernstein *et al*.'s transcription rate data [1] as the response variable in our study of the relationship between gene transcription and histone acetylation. These transcription rates were estimated by dividing the transcription levels by half-life time [16]. Due to concern that measured microarray data may vary significantly among different microarray platforms or research groups [32-34], we repeated our analysis using an independent dataset [15]. The results obtained from the two gene transcription data are similar (Additional data file 1).

After removing genes (with their corresponding intergenic and coding regions) that have missing data in any of the above datasets, we merged all the data into a single dataset, which contains 3,049 intergenic and 3,384 coding regions. The genomic sequence of *S. cerevisiae *was downloaded from the *Saccharomyces *Genome Database [14]. The promoter sequences (up to 800 base pairs (bp) upstream of the translation start site of each gene) were extracted for cis regulatory signal analysis.

### Delineating *cis *regulatory information

Transcription factors regulate genes by binding to transcription factor binding sites (TFBSs), which are short sequence segments (approximately 10 bp) located near genes' transcription start sites (TSSs). In yeast, these binding sites are mostly within 500 bp upstream of each gene's TSS. It has been shown that a gene's expression pattern can be predicted to a great extent by its upstream sequence information [26]. We took two different approaches to accommodate sequence information in our analysis of the histone acetylation effect.

In our first approach, we conducted *de novo *TFBS predictions using MDscan [24] among the upstream sequences of the genes that were transcribed at high rates [1]. In particular, this algorithm searched for enriched sequence motifs of widths 5 to 15 in the promoter sequences, resulting in 580 statistically significant, possibly overlapping, candidate TFBMs (*p *value < 0.05). We then used these motif patterns to scan all promoter regions for matches so as to compute a motif score for each TFBM at each promoter [24]. To avoid overfitting, we selected a subset of 33 functional motifs based on the association of the motif score of a promoter with the transcription rate of the corresponding gene. In particular, we used both a linear regression procedure, Motif Regressor [25], and a model-free method, regularized sliced inverse regression (RSIR) [35], as explained below.

Our second approach to account for the *cis *regulatory information was to directly use the 666 transcription factor binding motifs reported by Beer and Tavazoie [26], which is a combination of computational predictions using AlignACE [22] and 51 experimentally derived ones [36,37]. Since these motifs have been shown to have a high predictive power for gene expression patterns, they may also be informative for predicting transcription rate. Out of these 666 motifs, our linear regression and RSIR procedures (see below) found 15 that are highly relevant to predicting gene transcription rates.

### Model free motif selection

RSIR [35] is a statistical method for dimension reduction and variable selection. It assumes that gene *i*'s transcription rate *y*_*i *_and its sequence motif scores **x**_*i *_= (*x*_*i*1_,...,*x*_*iM*_)^*T *^are related as:

yi=f(β1Txi,β2Txi,...,βkTxiεi),     (equation 3)
 MathType@MTEF@5@5@+=feaafiart1ev1aaatCvAUfeBSjuyZL2yd9gzLbvyNv2Caerbhv2BYDwAHbqedmvETj2BSbqee0evGueE0jxyaibaiKI8=vI8tuQ8FMI8Gi=hEeeu0xXdbba9frFj0=OqFfea0dXdd9vqai=hGuQ8kuc9pgc9s8qqaq=dirpe0xb9q8qiLsFr0=vr0=vr0dc8meaabaqaciGacaGaaeqabaqadeqadaaakeaacaWG5bWaaSbaaSqaaiaadMgaaeqaaOGaeyypa0JaamOzaiaacIcaiiqacqWFYoGydaqhaaWcbaGaaGymaaqaaiaadsfaaaacbeGccaGF4bWaaSbaaSqaaiaadMgaaeqaaOGaaiilaiab=j7aInaaDaaaleaacaaIYaaabaGaamivaaaakiaa+HhadaWgaaWcbaGaamyAaaqabaGccaGGSaGaaiOlaiaac6cacaGGUaGaaiilaiab=j7aInaaDaaaleaacaWGRbaabaGaamivaaaakiaa+HhadaWgaaWcbaGaamyAaaqabaGccqaH1oqzdaWgaaWcbaGaamyAaaqabaGccaGGPaGaaiilaiaaxMaacaWLjaWaaeWaceaacaqGLbGaaeyCaiaabwhacaqGHbGaaeiDaiaabMgacaqGVbGaaeOBaiaabccacaaIZaaacaGLOaGaayzkaaaaaa@5CBB@

where *f*() is an unknown (and possibly nonlinear) function, **β**_*l *_= (*β*_*l*1_,...,*β*_*lM*_)^*T*^, (*l *= 1,...,*k*), are vectors of linear coefficients, and *ε*_*i *_represents the noise. The number *k *is called the dimension of the model. A linear regression model is a special one-dimensional case of equation 3. RSIR estimates both *k *and the **β**_*l *_values without estimating *f*(). Since many entries of the *β*_*lj *_values are close to zero, which implies that the corresponding motif scores contribute very little in equation 2, we retain only those motifs whose coefficient *β*_*lj *_is significantly nonzero.

We applied RSIR to the 580 candidate motifs selected by MDscan and the 666 motifs from [26], with the transcription rate as the response variable. In both cases, *k *was estimated as 1, and *f*() showed a strong linear pattern. We found 104 and 69 motifs, respectively, that have significantly nonzero coefficients in our RSIR model.

Previous application suggests that RSIR is conservative in selecting variables [35]. We applied the stepwise regression algorithm (which is a recursive method commonly used for variable selection; see page 347 of [27]) to further reduce the number of motifs. In the end, a total of 33 motifs from MDscan and 15 motifs from [26] were retained for further use. These motifs represent our summary of sequence-specific information on gene transcription rates.

### Model validation

To assess the significance of our model for controlling the confounding effects due to sequence information, we randomly permuted the transcription rates data 50 times and repeated the same statistical procedures: identifying motif candidates using MDscan, selecting the most significant motifs using RSIR, and fitting the linear regression model. The distribution of R^2 ^obtained for these randomized data was used as a baseline to evaluate the significance of our statistical procedure.

We also performed a five-fold cross validation procedure to test whether equation 2 overfit data. In particular, the full set of genes (seethe Data sources section above) was randomly partitioned into five subsets of equal sizes. Each subset was used for testing in turn with the rest used for training. For each training subset, the sequence motifs were inferred using MDscan, RSIR, and stepwise regression methods. We fit the model equation 2 using the training data and then evaluated out-of-sample error by applying to the testing data. The in-sample and out-of-sample root mean square errors were then compared.

### Partial correlation

Let *X *and *Y *represent two random variables and *Z *= (*Z*_1_,*Z*_2_,...,*Z*_*p*_) be a set of control random variables. The linear relationship between *X *and *Z *can be estimated via a linear regression model *X *= *α*_*X *_+ *Zβ*_*X *_+ *ε*_*X*_, similarly for that between *Y *and *Z*. The residues *ε*_*X *_and *ε *_*Y *_contain the information left unexplained by *Z*. The partial correlation between *X *and *Y *while controlling *Z *is defined as the Pearson correlation between *ε*_*X *_and *ε*_*Y*_.

## Additional data files

The following additional data are available with the online version of this paper: Additional data file 1 contains supporting text, figures, and tables. The adequacy of the linear regression, normalization of the Kurdistani *et al*. data, and sensitivity issues are discussed in further detail in the text. The figures and tables included demonstrate and compare the Pokholok *et al*. and Kurdistani *et al*. data.

## Supplementary Material

Additional File 1 Supporting text, figures, and tables relating to the Kurdistani *et al*., Pokholok *et al*. and Kurdistani *et al*. data. Click here for file

## Figures and Tables

**Table 1:** Model performance (adjusted R^2^) with different covariates

	Intergenic regions				Coding regions			
Acetylation sites included	-	Seq	Nuc	Seq/Nuc	-	Seq	Nuc	Seq/Nuc

-	0	0.1387	0.1145	0.1997	0	0.1315	0.1440	0.2185
H3K9 and H3K14	0.1808	0.2700	0.2641	0.3208	0.1014	0.2059	0.2515	0.3068
H4	0.0849	0.2086	0.2487	0.3085	0.0222	0.1522	0.2131	0.2774
H3K9, H3K14, and H4	0.1841	0.2706	0.2704	0.3262	0.1957	0.2627	0.2619	0.3131

The adjusted R^2 ^for the linear regression model (equation 2) containing different regulatory factors (Nuc, nucleosome occupancy; Seq, sequence information). (The adjusted R^2 ^is related to the (unadjusted) R^2 ^as Radjusted2=1−[(n−1)/(n−p−1)](1−Runadjusted2)
 MathType@MTEF@5@5@+=feaafiart1ev1aaatCvAUfeBSjuyZL2yd9gzLbvyNv2Caerbhv2BYDwAHbqedmvETj2BSbqee0evGueE0jxyaibaiKI8=vI8tuQ8FMI8Gi=hEeeu0xXdbba9frFj0=OqFfea0dXdd9vqai=hGuQ8kuc9pgc9s8qqaq=dirpe0xb9q8qiLsFr0=vr0=vr0dc8meaabaqaciGacaGaaeqabaqadeqadaaakeaacaWGsbWaa0baaSqaaiaadggacaWGKbGaamOAaiaadwhacaWGZbGaamiDaiaadwgacaWGKbaabaGaaGOmaaaakiabg2da9iaaigdacqGHsislcaGGBbGaaiikaiaad6gacqGHsislcaaIXaGaaiykaiaac+cacaGGOaGaamOBaiabgkHiTiaadchacqGHsislcaaIXaGaaiykaiaac2facaGGOaGaaGymaiabgkHiTiaadkfadaqhaaWcbaGaamyDaiaad6gacaWGHbGaamizaiaadQgacaWG1bGaam4CaiaadshacaWGLbGaamizaaqaaiaaikdaaaGccaGGPaaaaa@5992@, where *n *is the sample size, and *p *is the number of explanatory variables in the linear regression model.)

**Table 2:** Mean transcription rates (log-transformed) for genes with similar histone acetylation patterns

	H3K9ac Low	H3K9ac High
H3K14ac Low		
H4ac Low	-0.850	0.207
H4ac High	-0.522	0.307
		
H3K14ac High		
H4ac Low	-0.454	0.816
H4ac High	-0.126	0.460

Ac, acetylation.

**Table 3:** Partial correlation between covariate and transcription rates

	Intergenic regions				Coding regions			
Covariate	Control variable	Partial correlation	Control variables	Partial correlation	Control variable	Partial correlation	Control variables	Partial correlation

H3K9	H4	0.3015	H4 and Seq	0.2507	H4	0.2439	H4 and Seq	0.2038
H3K14	H4	0.2359	H4 and Seq	0.2105	H4	0.4070	H4 and Seq	0.3473
H4	H3K9, H3K14	-0.0656	H3K9, H3K14 and Seq	-0.0344	H3K9, H3K14	-0.3245	H3K9, H3K14 and Seq	-0.2678

The partial correlation between transcription rates and H3 (or H4) acetylation levels while controlling for the effects of H4 (or H3) acetylation and sequence information (Seq).
