# Supplementary material for: Statistical assessment of the global regulatory role of histone acetylation in Saccharomyces cerevisiae
Source: Genome Biol. 2006 Aug 2;7(8):R70. doi: 10.1186/gb-2006-7-8-r70 (PMC1779595; doi:10.1186/gb-2006-7-8-r70)

Statistical Assessment of the Global Regulatory Role of Histone Acetylation in *Saccharomyces cerevisiae*

(Support Information)

Authors: Guo-Cheng Yuan, Ping Ma, Wenxuan Zhong and Jun S. Liu

Linear Relationship between RSIR Selected Motifs and Transcription Rates

Variable selection using the RSIR algorithm does not assume a linear relationship. In principle, the functional relationship can be rather general, which is not explicitly estimated by the RSIR procedure. However, in our case, a linear relationship fits the data pretty well, since a linear regression model is highly statistically significant (*p*-value $< 5.4\times10^{-6}$) and the residuals do not show obvious nonlinear or non-Gaussian pattern. Such a relationship is also suggested by the scatter plot (Figure S1).

Statistical Significance of Quadratic Interaction Coefficients

In the main text, we showed that including quadratic interaction terms in Eqn (2) does not lead to significant changes of R-square. We also tested the statistical significance of the regression coefficients corresponding to the quadratic interaction terms (Table S1). Without controlling the confounding effect due to sequence motif or nucleosome occupancy information, the interaction between H3K9 and H3K14 is significant at the level of α = 0.05 (both for intergenic and coding regions). However, the significance of this interaction pair appears to be an artifact due to confounding, as all interaction terms are insignificant once the confounding effects are controlled (Table S1). These results further suggest that the cumulative model is justified.

Normalization of Kurdistani et al.'s Histone Acetylation Data

Kurdistani et al. [2] measured the acetylation level at eleven different sites. However, in their experiments, immunoprecipitated acetylated DNA was hybridized against genomic instead of nucleosomal DNA. As a result, the measured acetylation levels were confounded with variation of nucleosome occupancy. In order to remove this confounding effect, we normalized the raw acetylation data by fitting a linear regression model

$$x_{ij} = \alpha_j + \beta_j w_i + \varepsilon_{ij} \tag{S1}$$

where x_{ij} is the acetylation level at the j-th residue (log-transformed) and w_i is the nucleosome density [8] (log-transformed) at gene i. The residue ε_{ij} is retained as the *normalized acetylation value*. The linear correlation between acetylation levels and nucleosome occupancy is thus removed.

The validity of the above normalization method can be tested by comparing the normalized values with those measured by hybridizing acetylated DNA against nucleosomal DNA, which is not confounded by nucleosome occupancy. In fact, Pokholok et al. [8] measured the H3K14 acetylation levels using both methods, i.e., hybridizing histone acetylation against either nucleosomal or genomic DNA. These data serve as a good benchmark. By applying Eqn (S1) to the confounded H3K14 acetylation data, we found that the normalized data significantly improved the agreement with un-confounded data (Fig. S2). In particular, the Pearson correlation was increased from 0.79 to 0.86. More importantly, the confounded H3K14 acetylation data of Kurdistani et al. shows little correlation with transcription rates, which is clearly an artifact (Fig. S3a). After normalization, the correlation between the two was partially recovered (Fig. S3b). Taken together, the normalization procedure for removing the nucleosome occupancy bias significantly enhanced data quality. We also caution here, however, if the un-confounded acetylation data is intrinsically correlated with nucleosome

occupancy due to some unknown biological mechanisms, Eqn (S1) will become invalid since it will also remove this “intrinsic correlation”.

Sensitivity to Motif Finding Algorithms

In order to test whether our analysis is sensitive to motif finding, we repeated our analysis using an independent set of TF binding motifs and their matching scores and then applied Eqn (2). This second set of motifs (and their scores) was obtained from Beer and Tavazoie [26], who derived 666 TF binding motifs both by *de novo* search using AlignACE and from analyzing ChIP-chip experiments [36, 37]. We reduced this whole set of 666 motifs using our RSIR and stepwise regression procedure (see *Materials* and *Methods*). Only 15 motifs were eventually selected in our regression model. For comparison purposes, both our MDscan-based and literature-motif-based analyses were applied to the intersection of our merged dataset (containing 3049 intergenic and 3384 coding regions, see *Materials* and *Methods*) and Beer and Tavazoie’s dataset (after their tight-clustering pre-analysis, containing 1649 intergenic and 1816 coding regions). This subset contains 1468 intergenic and 1612 coding regions. Since the genes in Beer and Tavazoie’s dataset are pre-selected via a tight-clustering procedure so that they have well-defined expression patterns under different growth condition, it is not surprising that the performance of Eqn (2) when applied to this subset of genes is significantly better than to the whole gene set (compare Table S2 with Table 1), even for the same set of motif scores. Strikingly, about 20% of the variance can be explained by only 15 motifs alone, without additional acetylation or nucleosome occupancy information. Note that the two motif-based models give similar results (Tables S2 and S3), suggesting that our motif-based regression analysis is rather robust.

An interesting question is why the numbers of MDscan- and literature-based motifs differ substantially. First, computational methods may identify functionally redundant motifs as distinct ones, causing uncertainty in the

number of selected motifs. Second, the MDscan-based motifs were selected to explain genome-wide gene expression, whereas the literature-based motifs were chosen previously by other researchers based on a smaller set of genes which have “nice” gene expression patterns. Therefore, the MDscan-based motifs may also include those that have no regulatory role for this subset of genes. To eliminate irrelevant motifs, we selected a subset of MDscan-based motifs by stepwise regression, confining within the smaller gene set. As a result, 13 motifs were removed. Many of the remaining 20 motifs (Figure S4a) have patterns similar to the 15 motifs (Figure S4b) in the literature (Table S4), implying an intrinsic consistency between these two different motif-based methods. The slightly better performance of the literature-based motifs (Table S5) may be attributable to the authors' effort to optimize the “biological relevance” of their motifs using extra information, e.g., tight-clustering based on 255 microarray experiments, and additional experimentally verified motifs (based on CHIP-chip data).

Sensitivity to Variability among Different Genomic Data Sources

Even for identical biological samples, the measured microarray data still vary among different microarray platforms or research groups [32-34]. In the main text we presented results by analyzing the histone acetylation data in Pokholok et al. [8] and the gene expression data in Bernstein et al.[1]. To ensure robustness, we repeated our analysis using independent sources of histone acetylation [2] and gene expression data [15].

First, we tested reproducibility by replacing the acetylation data with those measured in Kurdistani et al. [2], which is normalized first as discussed above. For comparison, we selected the common set of intergenic and coding regions with our main dataset, resulting in a subset containing 1026 intergenic and 1492 coding regions. We repeated our analysis using both acetylation data on this subset of regions. The results are quite consistent with that presented in our main article (Tables S6 and S7), but more refined information can be gleaned from Kurdistani *et al.*'s data since they measured the acetylation level at eleven different acetylation sites.

Interestingly, the H2A and H2B acetylation levels have little correlation with transcription rates. The acetylation level at three different acetylation sites (K9, K18, and K27) on H3 tails are significantly correlated with gene expression, after controlling for the effects of acetylation at other histones. As suggested by the partial correlation analysis, H4 acetylation generally has little global regulatory effect except for H4K16, which seems to have a negative effect. However, we caution again for over-interpretation, as these results are based on the assumption that the un-confounded histone acetylation levels are uncorrelated with nucleosome density. The validity of this assumption requires further experimental tests.

Second, to test whether our results were affected by the variability of the gene expression data, we repeated our analysis using an independent gene expression dataset [15]. Notice that the microarray platforms in Holstege et al. and Bernstein et al. are different: Bernstein used spotted arrays, whereas Holstege used Affymetrix arrays. Again, we identified the subset of intergenic and coding regions in the two experiments. This subset contains 3049 intergenic and 3384 coding regions. The results from these two different gene expression datasets are similar (Tables S8 and S9).

Supplemental Tables

Table S1. The *p*-values for the quadratic interaction coefficients included in the linear regression model. (a) for intergenic regions; (b) for coding regions. (Nuc – nucleosome occupancy; Seq – sequence information).

(a)

**Interaction**	***p*-value for the interaction coefficient**			
	**—**	**Seq**	**Nuc**	**Seq Nuc**
**H3K9 and H3K14**	0.0012	0.1282	0.0229	0.2576
**H3K9 and H4**	0.7660	0.4647	0.4109	0.9322
**H3K14 and H4**	0.8790	0.2188	0.9761	0.4136

(b)

**Interaction**	***p*-value for the interaction coefficient**			
	**—**	**Seq**	**Nuc**	**Seq Nuc**
**H3K9 and H3K14**	0.0006	0.0314	0.8912	0.5015
**H3K9 and H4**	0.8908	0.7715	0.2486	0.1765
**H3K14 and H4**	0.7473	0.9758	0.2610	0.2252

Table S2. Comparison between the adjusted R-squares of the linear regression model using independently derived motif scores (*de novo* prediction using MDscan [24] vs directly from literature [26] to control sequence dependent regulatory effects based on the common sets of intergenic or coding regions. The number of motifs has been reduced by RISR and stepwise regression procedures. (a) for intergenic regions; (b) for coding regions.

(a)

Acetylation sites included	Model performance (adjusted R^2) with different covariates					
			MDscan motif		Literature motif	
	—	Nuc	Seq	Seq Nuc	Seq	Seq Nuc
—	0	0.1492	0.1950	0.2584	0.2132	0.2669
H3K9 and H3K14	0.2443	0.3295	0.3553	0.3975	0.3527	0.3904
H4	0.1342	0.3133	0.2949	0.3863	0.3016	0.3822
H3K9, H3K14, and H4	0.2460	0.3364	0.3549	0.4040	0.3522	0.3968

(b)

Acetylation sites included	Model performance (adjusted R^2) with different covariates					
			MDscan motif		Literature motif	
	—	Nuc	Seq	Seq Nuc	Seq	Seq Nuc
—	0	0.2431	0.1912	0.3174	0.1985	0.3128
H3K9 and H3K14	0.1666	0.3623	0.2847	0.4120	0.3048	0.4177
H4	0.0342	0.3224	0.2126	0.3794	0.2302	0.3854
H3K9, H3K14, and H4	0.2905	0.3694	0.3576	0.4173	0.3659	0.4216

Table S3. Comparison between partial correlation results using independently derived motif scores (*de novo* prediction using MDscan [24] and directly from literature [26] to control sequence dependent regulatory effects based on the common sets of intergenic or coding regions. The number of motifs has been reduced by RISR and stepwise regression procedures. (a) for intergenic regions; (b) for coding regions.

(a)

Covariate	Partial correlation between covariate and transcription rates					
	**Control variable**	**Partial correlation**	**Control variables**	**Partial correlation**	**Control variables**	**Partial correlation**
**H3K9**	H4	0.3201	H4 and Seq (MDscan)	0.2558	H4 and Seq (Literature motif)	0.2357
**H3K14**	H4	0.2811	H4 and Seq (MDscan)	0.2353	H4 and Seq (Literature motif)	0.2135
**H4**	H3K9, H3K14	-0.0543	H3K9, H3K14 and Seq (MDscan)	-0.0077	H3K9, H3K14 and Seq (Literature motif)	-0.0037

(b)

Covariate	Partial correlation between covariate and transcription rates					
	Control variable	Partial correlation	Control variables	Partial correlation	Control variables	Partial correlation
**H3K9**	H4	0.3386	H4 and Seq (MDscan)	0.2819	H4 and Seq (Literature motif)	0.2682
**H3K14**	H4	0.4952	H4 and Seq (MDscan)	0.4041	H4 and Seq (Literature motif)	0.3963
**H4**	H3K9, H3K14	-0.3862	H3K9, H3K14 and Seq (MDscan)	-0.3201	H3K9, H3K14 and Seq (Literature motif)	-0.2974

Table S4. The corresponding MDscan motif for each of the 15 literature motifs that are selected by RSIR.

Literature Motifs	MDscan motifs
Motif 1	Motif.N1.7.12
Motif 2	Motif.P1.15.1
Motif 3	Motif.P1.8.1
Motif 4	No good match
Motif 5	Motif.P1.7.1
Motif 6	Motif.P1.11.21
Motif 7	Motif N1.8.18
Motif 8	Motif.P1.11.21
Motif 9	Motif.P1.11.21
Motif 10	Motif.N1.12.28
Motif 11	Motif N1.8.18
Motif 12	Motif P1.7.1
Motif 13	Motif.P.1.15.1
Motif 14	Motif.P1.7.1
Motif 15	Motif.P1.7.1

Table S5. Same as Table S2 except for the MDscan-based motifs are further selected by a second round of stepwise regression.

(a)

Acetylation sites included	Model performance (adjusted R^2) with different covariates					
			MDscan motif		Literature motif	
	—	Nuc	Seq	Seq Nuc	Seq	Seq Nuc
—	0	0.1492	0.1909	0.2513	0.2132	0.2669
H3K9 and H3K14	0.2443	0.3295	0.3499	0.3886	0.3527	0.3904
H4	0.1342	0.3133	0.2910	0.3772	0.3016	0.3822
H3K9, H3K14, and H4	0.2460	0.3364	0.3494	0.3947	0.3522	0.3968

(b)

Acetylation sites included	Model performance (adjusted R^2) with different covariates					
			MDscan motif		Literature motif	
	—	Nuc	Seq	Seq Nuc	Seq	Seq Nuc
—	0	0.2431	0.1797	0.3117	0.1985	0.3128
H3K9 and H3K14	0.1666	0.3623	0.2770	0.4094	0.3048	0.4177
H4	0.0342	0.3224	0.2022	0.3760	0.2302	0.3854
H3K9, H3K14, and H4	0.2905	0.3694	0.3513	0.4144	0.3659	0.4216

Table S6. Comparison between the adjusted R-square for the linear regression model using different histone acetylation data (Pokholok et al. [8] and Kurdistani et al. [2]) based on the common sets of intergenic or coding regions. (a) for intergenic regions; (b) for coding regions..

(a)

Acetylation sites included	Model performance (adjusted R^2) with different covariates							
	Pokholok *et al.*				Kurdistani *et al.*			
	—	Seq	Nuc	Seq Nuc	—	Seq	Nuc	Seq Nuc
—	0	0.1169	0.1295	0.1918	0	0.1169	0.1295	0.1918
H2A and H2B	—	—	—	—	0.0120	0.1331	0.1386	0.2028
H3	0.1729	0.2494	0.2748	0.3184	0.1832	0.2541	0.3091	0.3383
H4	0.0928	0.1962	0.2671	0.3092	0.0693	0.1659	0.1982	0.2456
H3 and H4	0.1740	0.2491	0.2827	0.3243	0.2154	0.2765	0.3420	0.3679
H2A, H2B, H3 and H4	—	—	—	—	0.2201	0.2814	0.3461	0.3723

(b)

Acetylation sites included	Model performance (adjusted R^2) with different covariates							
	Pokholok *et al.*				Kurdistani *et al.*			
	—	Seq	Nuc	Seq Nuc	—	Seq	Nuc	Seq Nuc
—	0	0.1116	0.1225	0.1951	0	0.1116	0.1225	0.1951
H2A and H2B	—	—	—	—	0.0485	0.1466	0.1639	0.2269
H3	0.0579	0.1624	0.2058	0.2719	0.1917	0.2574	0.3071	0.3444
H4	0.0057	0.1208	0.1696	0.2424	0.0578	0.1576	0.1833	0.2460
H3 and H4	0.1479	0.2194	0.2169	0.2780	0.1911	0.2572	0.3075	0.3448
H2A, H2B, H3 and H4	—	—	—	—	0.2180	0.2776	0.3267	0.3601

Table S7. Comparison between partial correlation results using different histone acetylation data (Pokholok et al. [8] and Kurdistani et al. [2]) based on the common sets of intergenic or coding regions. (a) for intergenic regions; (b) for coding regions.

(a)

Covariate		Partial correlation between covariate and transcription rates			
		Control variable	Partial correlation	Control variables	Partial correlation
Pokholok et al.	H3K9	H4	0.2747	H4 and Seq	0.2233
	H3K14	H4	0.1919	H4 and Seq	0.1878
	H4	H3	-0.0483	H3 and Seq	-0.0244
Kurdistani et al.	H3K9	H2A, H2B, H4	0.2027	H2A, H2B, H4 and Seq	0.1883
	H3K14	H2A, H2B, H4	0.0570	H2A, H2B, H4 and Seq	0.0539
	H3K18	H2A, H2B, H4	0.3616	H2A, H2B, H4 and Seq	0.3284
	H3K23	H2A, H2B, H4	0.0834	H2A, H2B, H4 and Seq	0.0767
	H3K27	H2A, H2B, H4	0.1664	H2A, H2B, H4 and Seq	0.1430
	H4K8	H2A, H2B, H3	-0.0500	H2A, H2B, H3 and Seq	-0.0494
	H4K12	H2A, H2B, H3	-0.0350	H2A, H2B, H3 and Seq	-0.0292
	H4K16	H2A, H2B, H3	-0.2095	H2A, H2B, H3 and Seq	-0.1914
	H2AK7	H3, H4	0.0363	H3, H4 and Seq	-0.0020
	H2BK11	H3, H4	0.0483	H3, H4 and Seq	0.0051
	H2BK16	H3, H4	-0.0119	H3, H4 and Seq	-0.0336

(b)

Covariate		Partial correlation between covariate and transcription rates			
		Control variable	Partial correlation	Control variables	Partial correlation
Pokholok et al.	**H3K9**	H4	0.2234	H4 and Seq	0.2023
	**H3K14**	H4	0.3578	H4 and Seq	0.3123
	**H4**	H3	-0.3102	H3 and Seq	-0.2621
Kurdistani et al.	**H3K9**	H2A, H2B, H4	0.0510	H2A, H2B, H4 and Seq	0.0492
	**H3K14**	H2A, H2B, H4	0.0893	H2A, H2B, H4 and Seq	0.0901
	**H3K18**	H2A, H2B, H4	0.3167	H2A, H2B, H4 and Seq	0.2988
	**H3K23**	H2A, H2B, H4	0.0948	H2A, H2B, H4 and Seq	0.0966
	**H3K27**	H2A, H2B, H4	0.1034	H2A, H2B, H4 and Seq	0.0864
	**H4K8**	H2A, H2B, H3	0.0361	H2A, H2B, H3 and Seq	0.0445
	**H4K12**	H2A, H2B, H3	0.0056	H2A, H2B, H3 and Seq	0.0100
	**H4K16**	H2A, H2B, H3	-0.0184	H2A, H2B, H3 and Seq	-0.0120
	**H2AK7**	H3, H4	-0.0252	H3, H4 and Seq	0.0032
	**H2BK11**	H3, H4	-0.0062	H3, H4 and Seq	0.0046
	**H2BK16**	H3, H4	-0.0355	H3, H4 and Seq	0.0039

Table S8. Comparison between the adjusted R-squares for the linear regression model using different gene expression data sets (Bernstein et al. [1] and Holstege et al. [15]) based on the common sets of intergenic or coding regions. (a) for intergenic regions; (b) for coding regions.

(a)

Acetylation sites included	Model performance (adjusted R^2) with different covariates							
	Bernstein et al.				Hostege et al.			
	—	Seq	Nuc	Seq Nuc	—	Seq	Nuc	Seq Nuc
—	0	0.1387	0.1145	0.1997	0	0.1478	0.1101	0.2050
H3K9 and H3K14	0.1808	0.2700	0.2641	0.3208	0.1476	0.2445	0.2253	0.2892
H4	0.0849	0.2087	0.2487	0.3085	0.0548	0.1865	0.2042	0.2717
H3K9, H3K14, and H4	0.1841	0.2706	0.2704	0.3262	0.1525	0.2461	0.2289	0.2918

(b)

Acetylation sites included	Model performance (adjusted R^2) with different covariates							
	Bernstein et al.				Hostege et al.			
	—	Seq	Nuc	Seq Nuc	—	Seq	Nuc	Seq Nuc
—	0	0.1315	0.1440	0.2185	0	0.1408	0.1253	0.2124
H3K9 and H3K14	0.1014	0.2059	0.2515	0.3068	0.1221	0.2304	0.2519	0.3147
H4	0.0222	0.1522	0.2131	0.2774	0.0263	0.1639	0.1977	0.2714
H3K9, H3K14, and H4	0.1957	0.2627	0.2619	0.3131	0.2365	0.3050	0.2774	0.3340

Table S9. Comparison between partial correlation results using different gene expression data (Bernstein et al. [1] and Holstege et al. [15]) based on the common sets of intergenic and coding regions. (a) for intergenic regions; (b) for coding regions.

(a)

Covariate	Partial correlation between covariate and transcription rates							
	Bernstein et al.				Holstege et al.			
	Control variable	Partial correlation	Control variables	Partial correlation	Control variable	Partial correlation	Control variables	Partial correlation
**H3K9**	H4	0.3015	H4 and Seq	0.2507	H4	0.3133	H4 and Seq	0.2670
**H3K14**	H4	0.2359	H4 and Seq	0.2105	H4	0.1862	H4 and Seq	0.1471
**H4**	H3K9, H3K14	-0.0656	H3K9, H3K14 and Seq	-0.0344	H3K9, H3K14	-0.0782	H3K9, H3K14 and Seq	-0.0494

(b)

Covariate	Partial correlation between covariate and transcription rates							
	Bernstein et al.				Holstege et al.			
	Control variable	Partial correlation	Control variables	Partial correlation	Control variable	Partial correlation	Control variables	Partial correlation
**H3K9**	H4	0.2439	H4 and Seq	0.2038	H4	0.2544	H4 and Seq	0.2195
**H3K14**	H4	0.4070	H4 and Seq	0.3473	H4	0.4531	H4 and Seq	0.3989
**H4**	H3K9, H3K14	-0.3245	H3K9, H3K14 and Seq	-0.2678	H3K9, H3K14	-0.3613	H3K9, H3K14 and Seq	-0.3119

Supplemental Figures

Figure S1. Scatter plot of transcription rates vs the first RSIR covariates suggests linear relationship. All data are log-transformed.

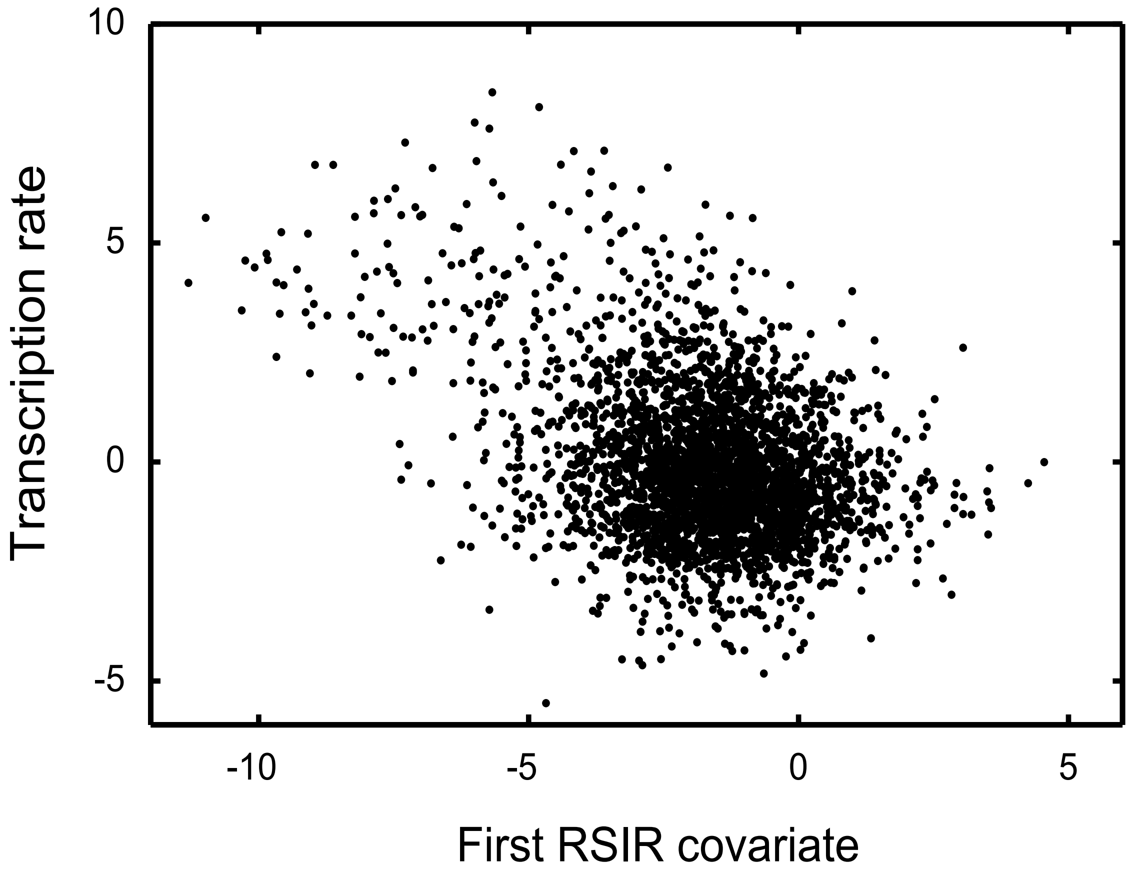

Figure S2. (a) Scatter plot of confounded (acevsWCE) vs un-confounded (acevsH3) H3K14 acetylation levels. (b) Scatter plot of normalized vs un-confounded H3K14 acetylation levels. Data obtained from Pokholok *et al.* (2005).

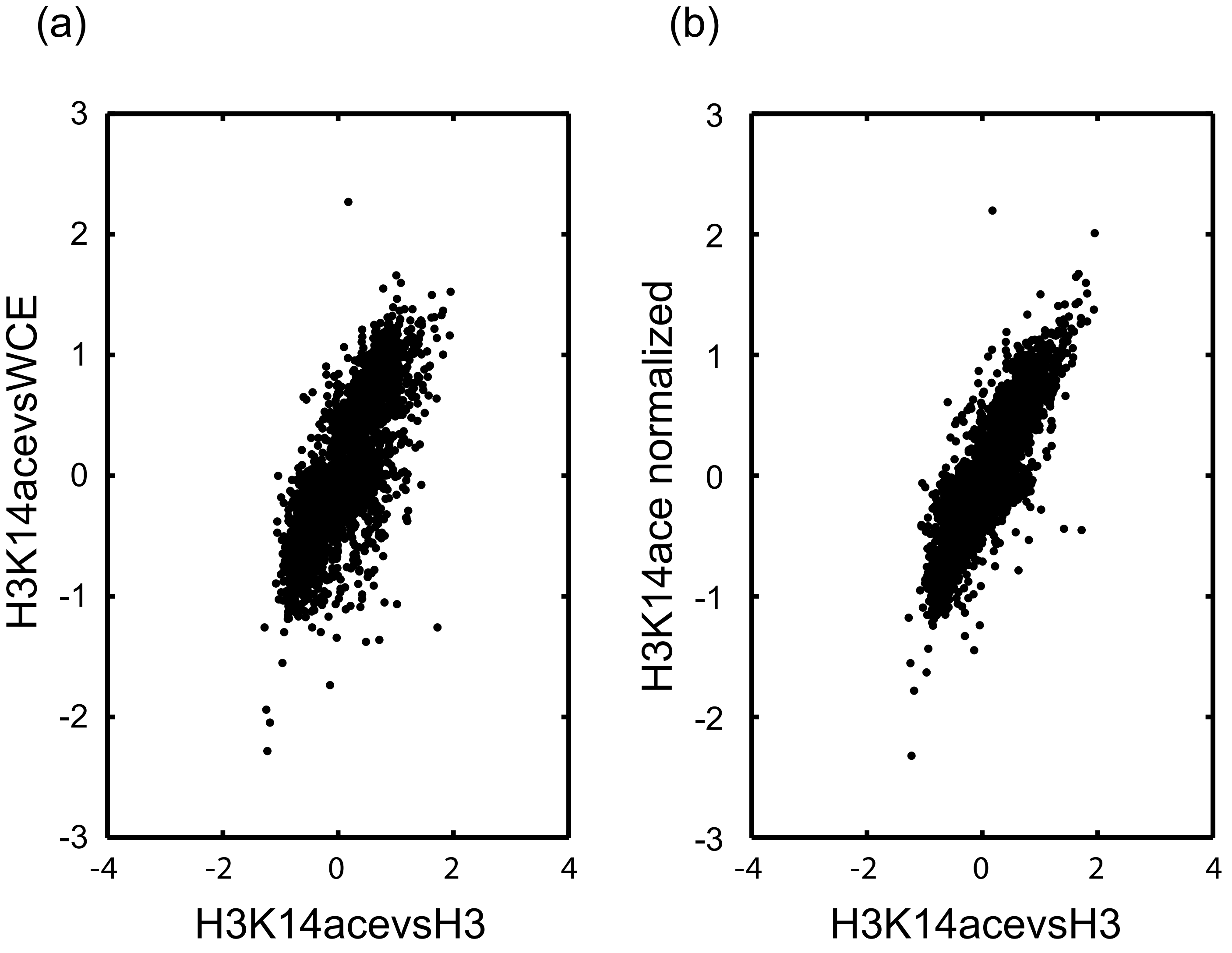

Figure S3. Normalization procedure partially recovers correlation between transcription rates and upstream H3K14 acetylation levels. (a) Transcription rates plotted against confounded (acevsWCE) and un-confounded (acevsWCE) H3H14 acetylation levels. (b) Trandscription rates plotted against normalized and un-confounded H3H14 acetylation levels.

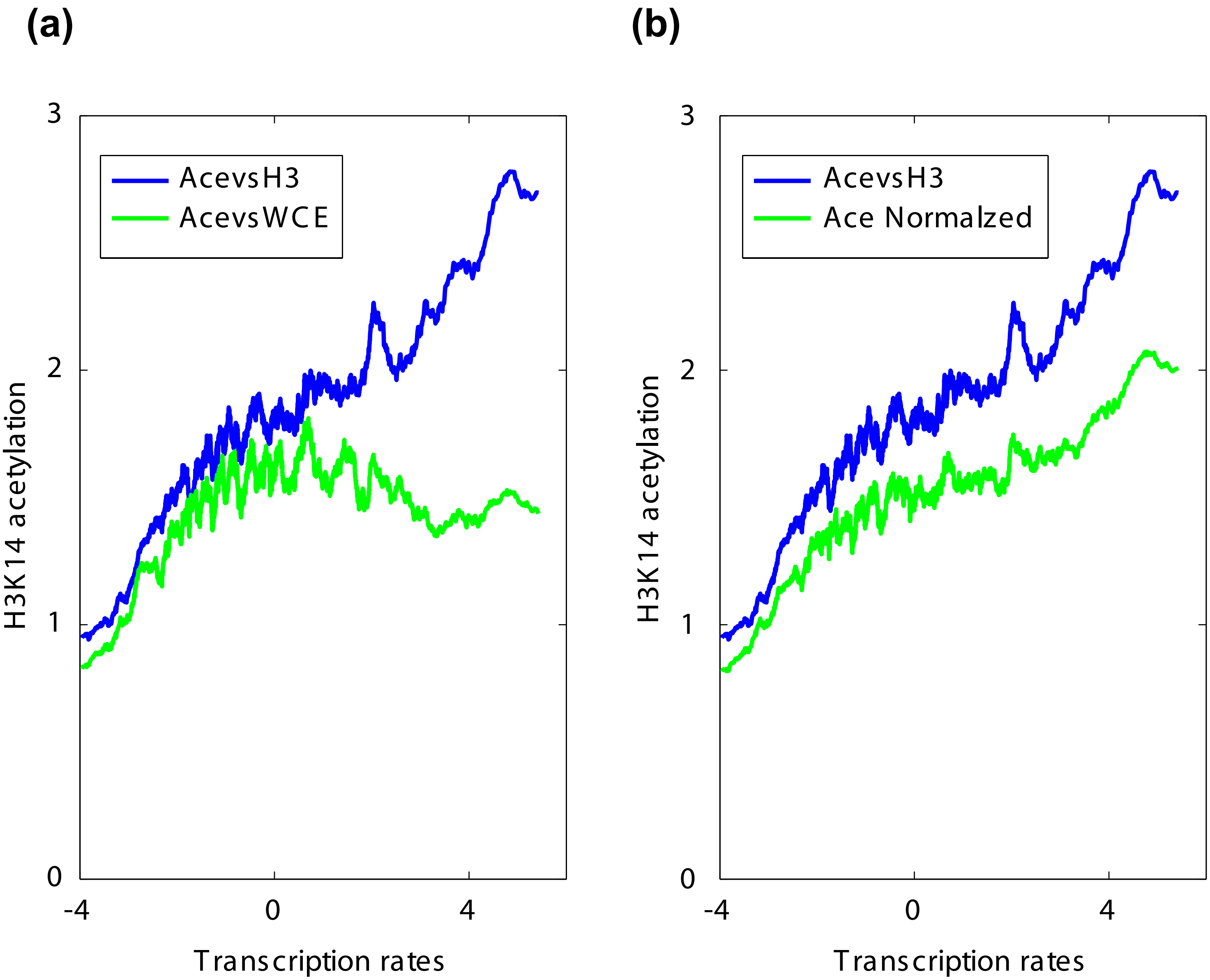

Figure S4. The motif patterns selected in the linear regression model. Motifs are obtained by MDscan (or literature) and further selected by RSIR and stepwise regression (see main text for details). (a) 20 MDscan motifs. (b) 15 literature motifs.

(a)

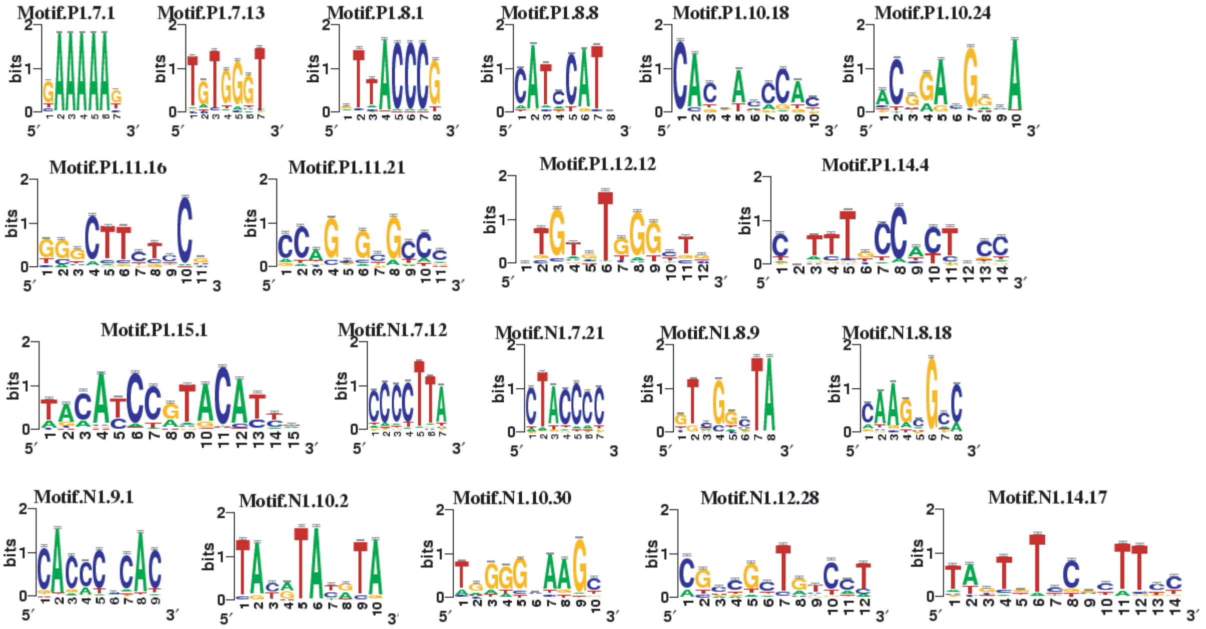

(b)

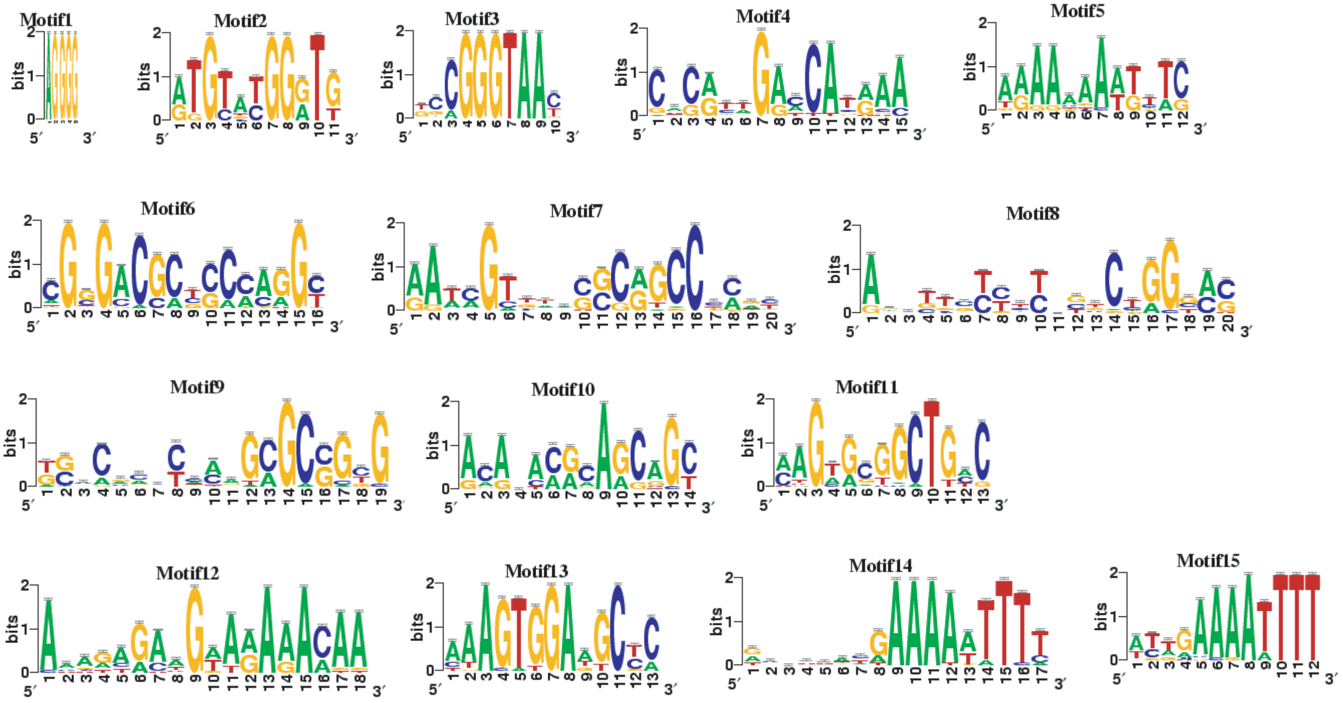

Supplement: Additional File 1 — Supporting text, figures, and tables relating to the Kurdistani et al., Pokholok et al. and Kurdistani et al. data. [file gb-2006-7-8-r70-S1.pdf]
